# Prevention and treatment of uncomplicated lower urinary tract infections in the era of increasing antimicrobial resistance—non-antibiotic approaches: a systemic review

**DOI:** 10.1007/s00404-019-05256-z

**Published:** 2019-07-26

**Authors:** Sara Wawrysiuk, Kurt Naber, Tomasz Rechberger, Pawel Miotla

**Affiliations:** 1 2nd Department of Gynaecology, Medical University of Lublin, ul. Jaczewskiego 8, 20-954 Lublin, Poland; 2 Department of Urology, Technical University of Munich, Munich, Germany

**Keywords:** Urinary tract infections, UTI, Non-antibiotic, Prevention, Treatment

## Abstract

**Purpose:**

Urinary tract infections (UTIs) are one of the more common infections encountered in everyday clinical practice. They account for 10–20% of all infections treated in primary care units and 30–40% of those treated in hospitals. The risk of UTI in the female population is considered to be 14 times higher than in the male population*.* The prevalence of bacterial etiology results in a large consumption of broad-spectrum antibiotics, which in turn leads to increased rates of resistant uropathogens. Therefore, non-antibiotic prevention and treatment options are now of great importance.

**Methods:**

A systematic literature search was performed for the last 20 years (1999–2019) and the efficiencies of these eight different non-antibiotic interventions were analysed and discussed.

**Results:**

This article provides an overview on non-antibiotic options for management of UTI, including the application of cranberry products, the phytodrug Canephron N, probiotics, nonsteroidal anti-inflammatory drugs (NSAID), d-mannose, estrogens, vitamins, and immunotherapy.

**Conclusions:**

The last 20 years of research on non-antibiotic approaches in UTI have not brought conclusive evidence that antibiotic usage can be replaced completely by non-antibiotic options. Hence, antibiotics still remain a gold standard for UTI treatment and prevention. However, changing the therapeutic strategy by including non-antibiotic measures in the management of UTI could be successful in avoiding antimicrobial resistance at least to some extent.

## Introduction

In everyday clinical practice, urinary tract infections (UTIs) are seen very frequently. They account for 10–20% of all infections treated in primary care units and 30–40% of those treated in hospitals [1]. The risk of UTI in the female population is considered to be 14 times higher than in the male population [2]. UTI can be divided into healthcare-associated urinary tract infections (HAUTIs) and community-associated UTIs (CAUTIs). CAUTI is commonly diagnosed in female patients with risk factors such as age, history of UTI, sexual activity and diabetes mellitus, whereas HAUTI is connected to medical interventions in the hospital. Herein, indwelling urinary catheters are seen as the main risk factors [3]. For gynecological patients, antibiotic prophylaxis of HAUTI is recommended before surgery for pelvic organ prolapse and/or stress urinary incontinence, abdominal or vaginal hysterectomy, laparoscopic hysterectomy and hysterosalpingography [4].

Recurrent UTIs (rUTIs) are another major problem in UTI management. Here, rUTI is defined as at least three UTI episodes occurring within 12 months, or at least two episodes happening within 6 months [5]. Patients with rUTI are often treated with antibiotics even for asymptomatic bacteriuria (ABU) associated with a higher occurrence of antibiotic resistance [6]. Long-term antibiotic treatment can also lead to alterations in the normal microbiome of the vagina and the gastrointestinal tract.

The most common uropathogens responsible for UTIs are *Escherichia coli* (with an incidence of around 65%), *Enterococcus faecalis, Klebsiella pneumonia* and *Proteus mirabilis* [7] with frequencies also depending on the kind of UTI, CAUTI or HAUTI. The antibiotic resistance rates in both CAUTI and HAUTI depend very much on the geographical location [3]. The prevalence of bacterial etiology in UTIs results in an extensive consumption of broad-spectrum antibiotics leading to increased rates of resistant uropathogens [8]. The frequent prescription of empirical antibiotics and the transmission of antibiotic-resistant genes, together with other resistance determinants of mobile genetic elements has finally led to the increased emergence of multidrug resistance (MDR) even at the community level, making UTI treatment even more difficult [9]. Studies have shown that development of resistance is very rapid, whereas its reversibility in clinical settings, as well as at the community level is slow [10]. Therefore, there is an urgent need to introduce new solutions in the management of UTI. Since extensive use of antibiotics for all kinds of infections has led to growing antibiotic resistance, non-antibiotic treatment options are now of great importance. Reducing the use of antibiotics will not only minimize antibiotic resistance, but also improve patient quality of life due to a smaller number of side effects coming about. This article provides an overview on eight different non-antibiotic options for preventing or treating UTIs between January 1999 and January 2019.

## Methods

A systematic literature search was performed of the 20 years period between January 1999 and January 2019 (inclusive) in PubMed (date of search February 2019) of articles on the efficiency of eight different non-antibiotic interventions for preventing and treating UTIs. These include cranberry products, the phytodrug Canephron N, probiotics, nonsteroidal anti-inflammatory drugs (NSAID), d-mannose, estrogens, vitamins and oral immunotherapy. The following key words were used: “UTI” or “urinary tract infections” AND “cranberry”, “canephron N”, “probiotics” or “lactobacillus”, “nonsteroidal anti-inflammatory drugs” or “NSAID”, “D-mannose”, “estrogens” or “ospemifene”, “vitamins” or “vitamin C” or “ascorbic acid” or “vitamin D”, “immunotherapy” or “urovaxom” or “immunisation” or “OM-89”, “UTI” or “urinary tract infections” AND “non-antibiotic” or/and “prevention” or “treatment”. Randomized controlled trials and observational studies were conducted on non-pregnant women, aged over 18 years, generally healthy with or without risk factors for rUTI. The interventions were compared to groups of female patients receiving placebo or antibiotic therapy. As outcome criteria, UTI symptoms and the incidence of bacteriuria were analysed and reported.

## Results

### Cranberry products

Cranberries in a form of juice or tablets are widely used and self-administered for prevention of UTI. Their mechanism of action includes inhibition of bacterial (mainly *E. coli*) adhesion to uroepithelial cells [11]. When adhesion is blocked, bacteria are not able to invade the mucosal surface of the urinary tract. In the study conducted by Singh et al. in a group of patients, after 12 weeks of receiving cranberry extracts, when compared to placebo, bacterial adhesion decreased. Cranberry extracts were also superior to placebo in terms of urine pH reduction and prevention of UTI symptoms such as dysuria, bacteriuria, and pyuria [12].

In the latest Cochrane Review, the authors concluded that cranberry juice did not decrease the number of UTIs and thus cranberry has no significant benefit in preventing UTI [13]. This was a major change to the previous Cochrane report [14], where some evidence was found that cranberry juice may decrease the number of symptomatic UTIs over a 12-month period, particularly for women with rUTIs. Its effectiveness for other groups was less certain. Since then, new clinical trials on cranberry products have been conducted. Foxman et al. assessed the therapeutic effects of cranberry juice and the risk of UTI after gynecologic surgery wherein the patient was catheterized. In this randomized, double-blinded, placebo-controlled trial, 160 patients received cranberry capsules or placebo. The results showed that in the cranberry treatment group, the incidence of UTI was significantly lower than in the placebo group. Furthermore, among women after elective benign gynecological surgery with catheter placement, the use of cranberry capsules reduced the risk of UTI during the postoperative period by half [15].

Maki et al. gauged the effect of cranberry juice consumption on the occurrence of UTI episodes in women with a recent history of UTI. For 24 weeks, 185 women received 240 ml of cranberry juice, while another 185 women were given a placebo beverage. The results showed that 1 in 3.2 incidences, clinical UTI was prevented through cranberry intervention [16]. Takahashi et al. also conducted a randomized, double-blinded study and demonstrated that cranberry beverage is superior to placebo in terms of UTI prevention, but this was only observed in a group of female patients over 50 years of age. They concluded that the efficiency of cranberry products still remains controversial since it reduced the risk of UTI only in a limited population [17]. In contrast, cranberry juice did not significantly reduce UTI risk compared with placebo in a study conducted by Stapleton et al. in which 176 premenopausal women with a recent history of UTI were randomized (120 to cranberry juice and 56 to placebo) and followed up for a median of 168 days, although a trend of protective effect was observed in this study [18].

The main substances responsible for the inhibition of adhesion of *E. coli* to the urinary mucosa are the proanthocyanidins of two types of linkage—A- and B-type. The protective abilities of Cranberry juice are due to proanthocyanidins of A-type [19]. Vostalova et al. suggested that with regard to active components in cranberry juices, the amounts are limited. They tested with a cranberry powder with a high concentration of proanthocyanidins (0.56%). Participants were randomly allocated to a cranberry (*n* = 89) or a placebo group (*n* = 93). The cranberry powder group which received 500 mg of cranberry for 6 months experienced a longer time to first UTI then did the placebo group. Moreover, the intent-to-treat analyses showed that in the cranberry group, the UTIs were significantly fewer [10.8% vs. 25.8%, *p* = 0.04, with an age-standardized 12-month UTI history (*p* = 0.01)] [20].

The overall results suggest that cranberry products may be an option for prevention of UTI in healthy, non-pregnant patients, as well as in patients after gynecological surgery when a catheter was placed. However, these findings still need confirmation because the conducted studies did not involve a large enough number of participants. What is more, Liska et al. in their meta-analysis on cranberries and UTIs stated that a number of publications may report conflicting conclusion due to the fact that recommendations are mainly directed to women with rUTI and that they include outcome from various populations which also can cause differences in the results [21].

### Herbal therapy with Canephron N

Another alternative, non-antibiotic approach to UTI treatment is the administration of herbal preparations. One product approved in many countries is Canephron N (Bionorica, Germany). This contains century herbs, lovage roots and rosemary leaves. It has diuretic, spasmolytic, anti-inflammatory, antibacterial and nephroprotective properties, and is also considered to be safe in both pregnancy and during breastfeeding [22].

Miotla et al. evaluated the efficiency of Canephron N in the prevention of UTI in high-risk female patients undergoing urodynamic studies (UDS). Therein, women with at least one risk factor for UTI received after UDS either 3 g of fosfomycin trometamol (FT) single dose or 5 ml of Canephron N taken orally three times daily for 1 week. There was no statistically significant difference in UTI incidence between the two groups. The authors concluded that prophylaxis of UTI after UDS with this phytodrug may be a good alternative to antibiotics administered after UDS in high-risk female patients [23].

Wagenlehner et al. conducted a double-blinded, multicenter, non-inferiority study comparing the efficiency of UTI treatment with Canephron N to standard antibiotic therapy with fosfomycin trometamol. A large cohort of female patients with symptoms of acute uncomplicated lower UTI was randomly allocated into two groups receiving either Canephron N 2 dragees t.i.d. or fosfomycin trometamol 3 g single dose with corresponding matched placebo. The results were very promising, as only 16.5% of patients treated with Canephron N required additional antibiotic treatment as compared to 10.2% in the fosfomycin group. The study, hence, demonstrated non-inferiority (inferiority margin 15% with 95% confidence interval) of the herbal compared to the antibiotic treatment. Of note, an additional advantage was that with Canephron N, fewer gastrointestinal side effects, such as diarrhoea and abdominal pain, were observed. In the phytodrug group, however, five episodes of pyelonephritis occurred (mainly during the first days of therapy), as compared to one episode of pyelonephritis in the fosfomycin group [24].

### Probiotics

The main argument in favour of using probiotics for UTI prevention and treatment is the fact that the vagina is a potential reservoir for the bacteria bringing about UTIs. Changes in the vaginal microbiota with reduction of protective *Lactobacillus *spp. colonies are associated with an increased risk of UTI. These changes can be induced by antimicrobial therapy, hormone level changes due to menopause or usage of contraceptives and even by UTI itself [25]. The use of either oral or intravaginal probiotics to restore the natural vaginal microbiota seems to be a promising approach to reducing antibiotic consumption and to decreasing antimicrobial resistance.

In a double-blind, non-inferiority trial conducted by Beerepoot et al. 252 postmenopausal women with rUTIs were randomized to receive 12 months of either antibiotic prophylaxis with trimethoprim-sulfamethoxazole or oral probiotic capsules containing 10^9^ colony-forming units of *Lactobacillus rhamnosus* GR-1 and *Lactobacillus reuteri* RC-14. The results of the study showed that supplementation with 480 mg *L. rhamnosus* GR-1 and L. reuteri RC-14 significantly decreased the mean number of recurrences in patients with uncomplicated UTIs when compared with trimethoprim-sulfamethoxazole administration. However, unlike trimethoprim-sulfamethoxazole, lactobacilli did not increase antibiotic resistance. In the study, after 1 month of trimethoprim-sulfamethoxazole prophylaxis, resistance to trimethoprim-sulfamethoxazole, trimethoprim and amoxicillin increased from approximately 20–40%, to approximately 80–95% in *E. coli* from the feces and urine of asymptomatic women and those with *E. coli*-induced UTIs [26].

In another double-blinded study, Stapleton et al. investigated premenopausal women with a history of rUTIs, by giving them daily either Lactin-V (*Lactobacillus crispatus* strains) or placebo for 5 days, then once weekly for 10 weeks. Results of this study showed a significant reduction of UTI episodes in patients who received intravaginal *Lactobacillus* treatment, compared to the placebo group. Herein, Lactin-V treatment resulted in prolonged colonization with *L. crispatus* strains and it reduced the frequency of rUTI by around 50% among participants. Unfortunately the results could not be compared directly with antibiotic prophylaxis of UTIs [27].

Lactobacilli may especially be useful for women with histories of recurrent, complicated UTIs or on prolonged antibiotic use. Probiotics are safe in terms of causing antibiotic resistance and may offer other health benefits due to vaginal re-colonisation with Lactobacilli. However, more comprehensive research is still needed before recommending probiotics as alternatives to antibiotics [28].

### Nonsteroidal anti-inflammatory drugs (NSAID)

The symptoms of UTIs are mostly connected to the inflammatory reaction of the urinary tract due to a significant increase in urinary prostaglandin production, because the onset and duration of clinical symptoms of UTI seem to be strongly connected to prostaglandin levels [29]. Since NSAIDs can inhibit the biosynthesis of prostaglandins [30], they can be useful in alleviating the symptoms of UTI. However, it is still not clear whether they can replace antibiotics in the treatment and/or prevention of UTIs.

In a double-blind, multicentre trial by Gagyor et al. female patients with UTI symptoms were randomized into groups receiving either fosfomycin trometamol (3 g single dose; 243 analysed) or ibuprofen (400 mg tid for 3 days; 241 analysed). Only patients with no risk and complicating factors were recruited. Herein, two-thirds of the ibuprofen group recovered without antibiotics, but had a significantly higher total burden of symptoms, while more had pyelonephritis (five cases in the ibuprofen and one in the fosfomycin group). The authors suggest that symptomatic treatment is a possible option to be considered in women with mild to moderate UTI symptoms [31]. Bleidorn et al. conducted a 6-months retrospective follow-up on this trial comparing antibiotics to NSAIDs and demonstrated no negative impact on patients in terms of recurrence and pyelonephritis after day 28 up to 6 months after trial participation [32]. In another randomized, double-blinded, non-inferiority trial involving 253 women with uncomplicated UTI, Kronenberg et al. compared the use of norfloxacin (*n* = 120; 400 mg bid) with diclofenac (*n* = 133; 75 mg bid) for 3 days. Here, fosfomycin trometamol was used as rescue antibiotic (3 g single dose) after completion of the study drug on day 3, if symptoms persisted. The study showed that diclofenac was inferior to norfloxacin in terms of symptoms relief, but it could reduce the usage of antibiotics, although it increased the risk of pyelonephritis (6 cases in the diclofenac and none in the norfloxacin group) [33]. Another randomized, controlled, double-blinded, non-inferiority trial conducted by Vik et al. compared the use of pivmecillinam (200 mg tid; 178 analysed) with ibuprofen (600 mg tid; 181 analysed) for 3 days in non-pregnant women with uncomplicated UTI. The study revealed that ibuprofen administration had some effect, albeit inferior to the use of pivmecillinum. The authors, however, concluded that ibuprofen cannot be recommended as initial treatment to women with uncomplicated UTIs until it is possible to identify patients that will develop complications, since all 7 patients who developed pyelonephritis received ibuprofen [34].

Replacing antibiotics with NSAIDs for the treatment of uncomplicated UTI may be at the cost of prolongation of symptoms and increased risk of pyelonephritis. Due to that fact, in uncomplicated UTI it is preferable to delay the use of antibiotic while closely monitoring the patient rather than completely resigning from antimicrobial treatment. In this strategy it is also important to share a decision-making process with a patient and to know his/her expectations towards the treatment [33]. Of note, NSAIDs have been studied only as a treatment option for UTIs, but not for prevention of rUTIs.

### d-Mannose

The urinary tract mucus membrane is coated with proteins that interfere with the adhesion of bacteria [5]. d-Mannose is a monosaccharide that can be rapidly absorbed and excreted by the urinary tract and can prevent the adhesion of type 1 bacterial fimbria to the uroepithelium. This is a bacterial virulence factor promoting UTI—especially caused by *E. coli* [35].

Kranjcec et al. randomly allocated female participants with rUTIs to three groups. The first group received 2 g of powdered d-mannose daily (*n* = 103), the second group got 50 mg of nitrofurantoin daily (*n* = 103) and the third group did not receive any prevention (*n* = 102) for 6 months. The results of the study showed that the risk of UTI was significantly reduced by both d-mannose and nitrofurantoin, with a lower risk of side effects in the d-mannose group. The authors conclude that d-mannose may be useful for UTI prevention, although more research is still required [36].

### Estrogens

Estrogen is a hormone not only responsible for regulating the female reproductive system, but also for stimulating the proliferation of lactobacilli, reducing vaginal pH and decreasing vaginal *Enterobacteriaceae* colonization [5]. Estrogen enhances the antimicrobial capacity of the uroepithelium, it inhibits bacterial multiplication and by strengthening the epithelial integrity, it prevents bacteria from reaching deeper levels of the uroepithelium [37] Stimulating these mechanisms is especially beneficial for post-menopause women with low estrogen levels who are afflicted with rUTIs. Hence, it is suggested that the use of topical estrogens can reduce the risk of recurrent infections. However, the efficiency of estrogens in UTI prevention still remains disputable.

Current literature shows both the reduction of UTI incidence after topical intravaginal estrogen use [38, 39] and failure in restoring vaginal microbiota and lowering UTI risk [40–42]. In the 2008 Cochrane Review on estrogens for preventing rUTIs in postmenopausal women, the authors concluded that vaginal estrogens reduced the number of UTIs in postmenopausal women with rUTI, but the results depended on the type of estrogen and the duration of treatment [43]. No new clinical trials on vaginal estrogens have been conducted within the last years. Moreover, oral estrogens are considered to be ineffective in UTI prevention and they are connected to a vast number of adverse effects such as breast tenderness or vaginal bleeding [44]. However, recently, an oral estrogen agonist/antagonist, ospemifene, was introduced. It is intended to be used to treat moderate-to-severe dyspareunia due to vulvovaginal atrophy and it has no major adverse effects on the breast, bone or cardiovascular systems of patients [44]. Hence, ospemifene could be a promising new non-antibiotic option for UTI prevention in postmenopausal patients, especially in those with additional vulvovaginal atrophy [45]. However, no valid data on its possible preventive effect are available at present. Further well-structured research on the use of ospemifene in rUTI is, therefore, needed.

### Vitamins

Vitamin C (ascorbic acid) supplementation is often recognized as a non-antibiotic prophylaxis for rUTIs. It has two suggested mechanisms of action. The first is urine acidification [46] and the second is a bacteriostatic effect mediated by the reduction of urinary nitrates to reactive nitrogen oxides [47]. However, evidence on vitamin’s C actual protective value on UTI in non-pregnant patients is limited and therefore its use should not be promoted.

Vitamin D is also recommended as a supplement for rUTI prevention. This is based on its function as an inducer of antibacterial innate immune responses [48]. Jorde et al. conducted a 5-year randomized study on both male and female participants. Five hundred and eleven subjects with prediabetes were randomly allocated to vitamin D3 (20,000 IU per week) versus placebo for 5 years. Therein, 116 patients received vitamin D and 111 were in the placebo group. Only 18 patients in the vitamin D group reported UTI, compared to 34 subjects in the placebo group. The effect on UTI was most pronounced in males. The authors suggest that supplementation with vitamin D might prevent UTI, but confirmatory studies are needed [49].

### Immunotherapy

Bacterial extracts are used in the management of UTI because they are able to stimulate the host’s immune system through the activation of monocyte-derived dendritic cells to produce antibodies and cytokines [50]. They may be an attractive option for prevention of UTI as they can be an alternative to antibiotic prophylaxis in patients with rUTIs.

In their review and meta-analysis, Naber et al. identified 11 studies which looked into the use of both oral and vaginal bacterial lysates in the prophylaxis of rUTIs [51]. Three out of four studies dealing with a vaginal vaccine were analysed (220 patients). The results showed the effectiveness of vaginal vaccine only when administered with a booster cycle (no rUTIs in 50% vs. 14% with placebo); however, adequate phase III studies are still missing. Seven of the studies dealt with an oral immunostimulant (OM-89), a lyophilized preparation of membrane proteins from 18 different uropathogenic *E. coli* (UPEC) strains [52]. Five out of seven studies were chosen for the meta-analysis. About 1000 female patients were analysed for an observation period of 6–12 months [53–56]. Accordingly, the mean number of UTI occurrence, as well as the use of antibiotics, was significantly lower in OM-89-treated group of patients in all the trials taken into the analysis (mean 39%) [51].

Aziminia et al. also systematically reviewed the evidence regarding the efficacy of vaccines or immunostimulants in reducing the recurrence rate of UTIs. They included five studies using OM-89. In four of these studies, a significant reduction of rUTIs was demonstrated [53–56]. In one later study [57] using a modified product (OM-89S) that is no longer available, the same results could not be confirmed. According to Aziminia et al. the lack of effect was probably due to the low number of UTIs during the study, the high number of protocol violations, and/or the modified manufacturing process.

Of another vaccine preparation, Urovac, consisting of ten heat-killed uropathogenic species—including six serotypes of *E. coli, Proteus vulgaris, Klebsiella pneumoniae, Morganella morganii,* and *Enterococcus faecalis*, three randomised, clinical trials were analysed. Therein, one study (*n* = 91) used high-dose vaccine, low-dose vaccine and placebo [58]; whereas two studies (*n* = 54 and *n* = 75) used vaccine with booster, without booster and placebo [59, 60]. Overall, Urovac reduced the risk of UTI recurrence for a half a year post-administration (RR 0.75, 95% CI 0.63–0.89; low QOE). This effect appears more pronounced in patients receiving vaccine with booster, compared to those receiving vaccine alone. Confirmation by larger phase-III studies by independent investigators is recommended.

In contrast to the aforementioned results, Huttner et al. conducted a randomized, single-blind, placebo-controlled phase 1b trial on 188 healthy female patients with rUTIs. The aim was to assess the safety and immunogenicity of *E. coli* O-antigen in the form of an intramuscular biconjugate vaccine containing *O*-antigens of four *E. coli* serotypes (ExPEC4V). The vaccine was well tolerated and there were no serious adverse effects in the experimental group. It also gave a functional antibody response that resulted in significantly fewer UTIs by different *E. coli* serotype in the vaccine group, compared with the placebo group. The evidence from this trial suggests that ExPEC4V did not reduce UTI recurrence compared to placebo at study endpoint (RR 0.82, 95% CI 0.62–1.10; low QOE), but the number of UTIs caused by different serotypes of *E. coli* was significantly lower in the vaccine group compared with the placebo group (0.207 mean episodes vs. 0.463 mean episodes; *p* = 0.002). Huttner et al. have now initiated phase II studies to confirm these promising findings [61].

Uromune is another bacterial extract with a potential benefit when used in UTI management. It is a sublingual spray composed of inactivated bacteria—*Escherichia coli, Klebsiella pneumoniae, Proteus vulgaris, and Enterococcus faecalis.* The mechanism of action of Uromune is based on a theory that stimulation of the sublingual mucosa will lead to an activation of an immune response in the urinary tract mucosa [62]. In Yang’s study, 75 female patients with rUTIs completed 3 months of Uromune therapy as a sub-lingual spray once a day. During the 12 month follow-up period, no UTIs were observed in 59 patients (78%). Out of 16 women who experienced UTI recurrence, 14 (87%) were postmenopausal. Currently, an international double-blind randomized control trial with Uromune versus placebo is being held [62].

For other bacterial lysates recommended for prophylaxis of rUTI, such as StroVac^®^ and SolcoUrovac^®^ containing ten heat-killed uropathogenic bacteria, Urostim^®^ containing four microbial species, and Urvakol^®^ containing 5 Gram-negative species and for adjuvant activity *Propionibacterium acnes*, no trials complying with the predefined criteria could be found within this period of time.

## Conclusions

UTIs are a common problem for women at different ages. Yet, UTIs are not only a problem unique for the patient, but are also a factor of high cost for the health care system. Increasing antimicrobial resistance with its expenditure and health consequences has raised interest in applying different non-antibiotic ways of preventing and treating uncomplicated lower UTIs. Unfortunately, the last 20 years of research on non-antibiotic approaches in UTI have not brought conclusive evidence that antibiotic usage can be replaced completely by non-antibiotic options. Hence, antibiotics still remain a gold standard for UTI treatment and prevention. However, changing the therapeutic strategy by including non-antibiotic measures in the management of UTI could be successful in avoiding antimicrobial resistance at least to some extent. According to the 2019 updated guidelines of the European Association of Urology, the prevention of rUTI includes first of all counseling of risk factors avoidance, then non-antimicrobial measures and finally antimicrobial prophylaxis. These interventions should be incorporated with an emphasis on that order [63]. With the proper identification of UTI risk factors, such as gender, prior UTIs, vaginal infection, sexual activity, the use of spermicidal agents, trauma/manipulation, diabetes mellitus, obesity and anatomic abnormalities [64], together with non-antibiotic interventions, a significant reduction of rUTI could probably be achieved, leaving only a few patients in whom antibiotic prophylaxis needs to be done as last resort. Although treatment of uncomplicated UTI with different NSAIDs was overall still inferior to antibiotic therapy, the strategy of treating as in other primarily benign bacterial diseases, primarily the host and not the bacteria, may stimulate further research for better alternatives. It should be particularly noted that the clinical outcome of uncomplicated UTI treated with the phytodrug Canephron N was not inferior to antibiotic therapy and showed comparable reduction of symptoms to antibiotic therapy. However, well-designed, randomized trials are still required to determine better the benefit and risk of non-antibiotic options for preventing and treating uncomplicated lower UTIs.
